# Repeatability of quantitative ^18^F-FLT uptake measurements in solid tumors: an individual patient data multi-center meta-analysis

**DOI:** 10.1007/s00259-017-3923-x

**Published:** 2018-01-23

**Authors:** G. M. Kramer, Y. Liu, A. J. de Langen, E. P. Jansma, I. Trigonis, M.-C. Asselin, A. Jackson, L. Kenny, E. O. Aboagye, O. S. Hoekstra, R. Boellaard, on behalf of the QuIC-ConCePT consortium

**Affiliations:** 1 Department of Radiology and Nuclear Medicine, VU University Medical Center, PO Box 7057, 1007 MB Amsterdam, Netherlands; 2 European Organisation for Research and Treatment for Cancer (EORTC), Headquarters, Brussels, Belgium; 3 Department of Pulmonology, VU University Medical Center, PO Box 7057, 1007 MB Amsterdam, Netherlands; 4 Medical Library, VU University Medical Center, PO Box 7057, 1007 MB Amsterdam, The Netherlands; 5 Division of Informatics, Imaging and Data Sciences Institute of Population Health, Wolfson Molecular Imaging Centre, Manchester Academic Health Sciences Centre, The University of Manchester, Manchester, UK; 6 Department of Surgery and Cancer, Imperial College London, Hammersmith Campus, London, UK

**Keywords:** Flt, Repeatability, PET, Oncology

## Abstract

**Introduction:**

3′-deoxy-3′-[^18^F]fluorothymidine (^18^F–FLT) positron emission tomography (PET) provides a non-invasive method to assess cellular proliferation and response to antitumor therapy. Quantitative ^18^F–FLT uptake metrics are being used for evaluation of proliferative response in investigational setting, however multi-center repeatability needs to be established. The aim of this study was to determine the repeatability of ^18^F–FLT tumor uptake metrics by re-analyzing individual patient data from previously published reports using the same tumor segmentation method and repeatability metrics across cohorts.

**Methods:**

A systematic search in PubMed, EMBASE.com and the Cochrane Library from inception-October 2016 yielded five ^18^F–FLT repeatability cohorts in solid tumors. ^18^F–FLT avid lesions were delineated using a 50% isocontour adapted for local background on test and retest scans. SUV_max_, SUV_mean_, SUV_peak_, proliferative volume and total lesion uptake (TLU) were calculated. Repeatability was assessed using the repeatability coefficient (RC = 1.96 × SD of test–retest differences), linear regression analysis, and the intra-class correlation coefficient (ICC). The impact of different lesion selection criteria was also evaluated.

**Results:**

Images from four cohorts containing 30 patients with 52 lesions were obtained and analyzed (ten in breast cancer, nine in head and neck squamous cell carcinoma, and 33 in non-small cell lung cancer patients). A good correlation was found between test–retest data for all ^18^F–FLT uptake metrics (R^2^ ≥ 0.93; ICC ≥ 0.96). Best repeatability was found for SUV_peak_ (RC: 23.1%), without significant differences in RC between different SUV metrics. Repeatability of proliferative volume (RC: 36.0%) and TLU (RC: 36.4%) was worse than SUV. Lesion selection methods based on SUV_max_ ≥ 4.0 improved the repeatability of volumetric metrics (RC: 26–28%), but did not affect the repeatability of SUV metrics.

**Conclusions:**

In multi-center studies, differences ≥ 25% in ^18^F–FLT SUV metrics likely represent a true change in tumor uptake. Larger differences are required for FLT metrics comprising volume estimates when no lesion selection criteria are applied.

**Electronic supplementary material:**

The online version of this article (10.1007/s00259-017-3923-x) contains supplementary material, which is available to authorized users.

## Introduction

Despite the recent progress made in cancer diagnosis and treatment, cancer remains the number one cause of death in the Western world [1]. Although treatment can be very effective, most regimens fail for a substantial number of patients. Early response evaluation enables the treating physician to differentiate responders from non-responders, to stop the treatment in the non-responder cohort timely and reliably. This potentially helps to limit side effects of anticancer therapies and avoid treatment delay of subsequent lines, thereby reducing patient burden and healthcare costs.

Several imaging modalities can be used to non-invasively assess response to treatment. Most modalities only evaluate morphological features, yet slow changes in tumor morphology or even pseudoprogression, as can be seen in case of immunotherapy, impair the use of morphological features in early repsonse assessment [2, 3]. However, morphological changes are often preceded by changes in tumor metabolism [4]. These early functional changes can be assessed using molecular imaging techniques such as PET, which may allow for more accurate early response evaluation.

There are several different radiotracers available to assess a variety of metabolic processes. One of these tracers is 3′-deoxy-3′-[^18^F]fluorothymidine (^18^F–FLT) and provides a method to evaluate cellular proliferation. Proliferation is a central hallmark of tumor growth and previous studies have validated ^18^F–FLT against the immunohistochemistry proliferation marker Ki67 in pathological specimens for several tumor types [5–7]. Unfortunately, ^18^F–FLT PET did not improve tumor detection or staging compared to 2-deoxy-2-[^18^F]fluoro-d-glucose (^18^F–FDG) due to lower sensitivity [8]. As proliferation is more cancer-specific compared to glycolysis, ^18^F–FLT PET has potential as an imaging biomarker for response assessment.

Cytotoxic and cytostatic therapies aim, respectively, to kill tumor cells (mainly highly proliferating cells) and diminish tumor growth, both leading to a decrease in cellular proliferation. After initiation of any antitumor treatment, this change in proliferation can be evaluated using ^18^F–FLT PET/CT. Several studies have been performed investigating ^18^F–FLT PET/CT as quantitative imaging biomarker of response [9], nevertheless most did not take variability into account.

For ^18^F–FDG, the repeatability of quantitative uptake measures has been widely investigated [10–13] and integrated into the response assessment criteria PERCIST [2]. Up to now, repeatability of quantitative ^18^F–FLT PET/CT has only been studied in a few small single-center cohorts (≤ 10 patients) [14–17]. Moreover, there was variability in uptake intervals, tumor delineation methods, and image analyses. The aim of this study was therefore to perform an individual patient data meta-analyses by re-analyzing all available ^18^F–FLT repeatability data from previously published studies and to determine the repeatability of several quantitative ^18^F–FLT tumor uptake metrics using similar uptake intervals, the same tumor segmentation method, and the same repeatability metrics as would be done in a prospective multi-center study.

## Methods

### Search strategy and selection process

To identify all relevant publications, a systemic search was performed in PubMed, EMBASE.com and the Cochrane Library (via Wiley) from inception to October 20, 2016 (last elicitation). A combination of the search terms comprising ‘FLT-PET’ and ‘neoplasms’ was used. This included MeSH terms and controlled terms from EMtree for PubMed and EMBASE.com, respectively, as well as free-text terms. We only used free-text terms in the Cochrane Library (see supplemental data). All potentially relevant titles and abstracts were screened for eligibility. Full-text articles were checked for eligibility criteria where necessary. References of eligible publications were checked for relevant publications. We have also checked ClinicalTrials.gov and The European Union Clinical Trials Register for ongoing and unpublished studies.

Studies were included if they met the following criteria:

- The study investigated the repeatability of ^18^F–FLT PET or PET/CT in oncological patients;
- Scans were performed on two separate days using the same scanner; and
- Patients were not treated in between both scans.

Studies were excluded if they met the following criteria:

- Animal or in vitro studies;
- Focused on tumors of the central nervous system (to avoid differences in pharmacokinetics due to the blood–brain barrier);
- Not available in full text or not written in English; and
- Reviews, editorials, letters, legal cases, interviews, case reports, and comments.

### Data analysis

Sites from all identified cohorts were contacted, and permission was requested to re-analyze the original ^18^F–FLT PET repeatability scans. All datasets consisted of 60- or 95-min dynamic test and retest ^18^F–FLT PET scans. Where permission was granted, original ^18^F–FLT scans of all individual patients were supplied in DICOM or Analyze format. Prior to re-analysis, all scans were checked for technical issues and artifacts. If any technical issues or artifacts were present, data were cross-checked with the original research teams. After checking of the scan data, static standard uptake value (SUV) images were generated from the dynamic images: 40–65 or 45–60 min post-injection, depending on the original frame definition. A 5-mm Gaussian filter was applied to the non-smoothed reconstructed images to match the spatial resolution between existing datasets and with previously published data. New volumes of interest (VOI) were defined by segmenting tumors using a 50% isocontour of the SUV_peak_ (1.2 cm in diameter sphere positioned to maximize its mean value), adapted for local background (in-house developed software) [12, 18]. For each VOI, SUV_max_, SUV_mean_, SUV_peak_, proliferative volume (50% threshold of SUV_peak_ corrected for local background) and total lesion uptake (TLU, product of SUV_mean_ and proliferative volume) was determined. These quantitative ^18^F–FLT uptake metrics were checked for outliers and discrepancies with the original data, however no important issues were identified. In addition, tumor-to-blood ratios (TBR) were calculated by normalizing tumor SUVs to the bloodpool SUV_mean_ of a large vascular structure (2 × 2 voxel VOI in five consecutive planes) [19]. ^18^F–FLT uptake in the tumor was normalized to the SUV_mean_ of the carotid artery in HNC data and to the ascending aorta for all other lesions. All SUVs were calculated by normalizing the radioactivity concentrations by the injected ^18^F–FLT dose and body weight and were corrected for physical decay.

### Statistical analysis

Repeatability of the quantitative uptake and volume metrics was determined by calculating the mean and standard deviation (SD) of the percentage differences between the two baseline scans:

1 $$ \% Difference=\frac{Scan\ 2- Scan\ 1}{\left( Scan\ 1+ Scan\ 2\right)/2}\times 100 $$

Normality of the data was assessed using histogram analyses and quantile-quantile plots (data not shown). The repeatability coefficients (RC) were calculated as 1.96 × SD of the percentage differences. A paired *t* test was performed to test for significant differences in mean uptake between both baseline scans. To assess the significance of differences in RC, the Levene’s test was used. Moreover, the intra-class correlation coefficient (ICC) using a two-way mixed model, model II regression analysis [20] and Bland–Altman plots were used to evaluate correlations and biases between the test-and-retest scans. The effect of various lesion selection strategies on repeatability was evaluated: lesions ≥ 4.2 ml (diameter ≥ 20 mm) [18], SUV_max_ ≥ 4.0 [10, 11], hottest lesion per scan (highest SUV_max_) or primary lesions only. In addition, the uptake values of individual lesions were averaged per patient to obtain the averaged uptake and assess repeatability on a patient level. All statistical analyses were performed using SPSS 22.0 (SPSS, Chicago, IL, USA).

## Results

### Search results

**Fig. 1:**
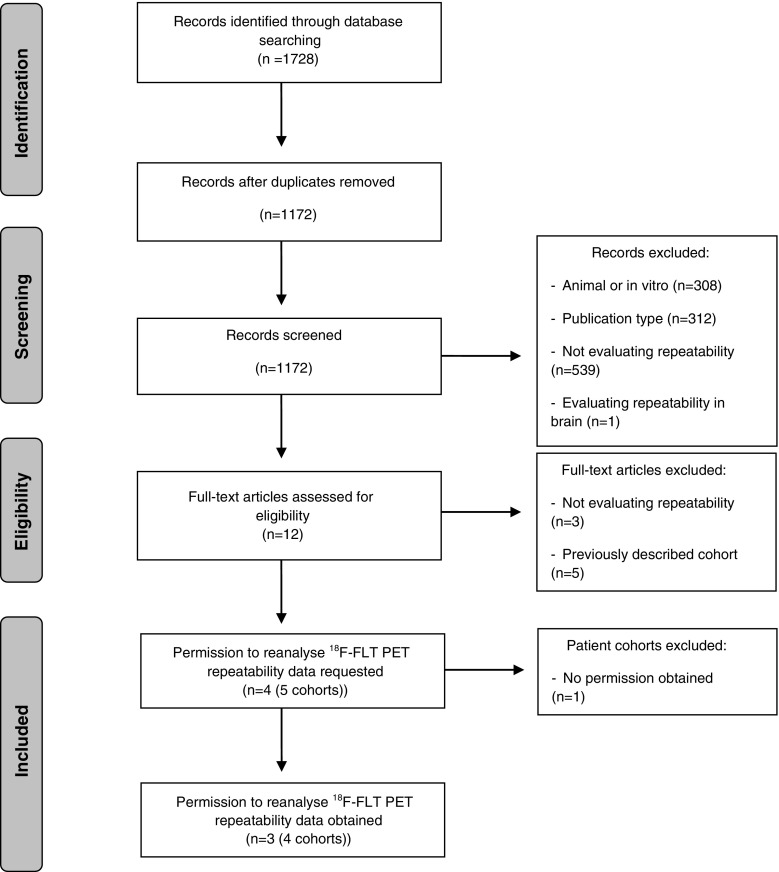
Flowchart of the search-and-selection procedure of studies

**Fig. 2:**
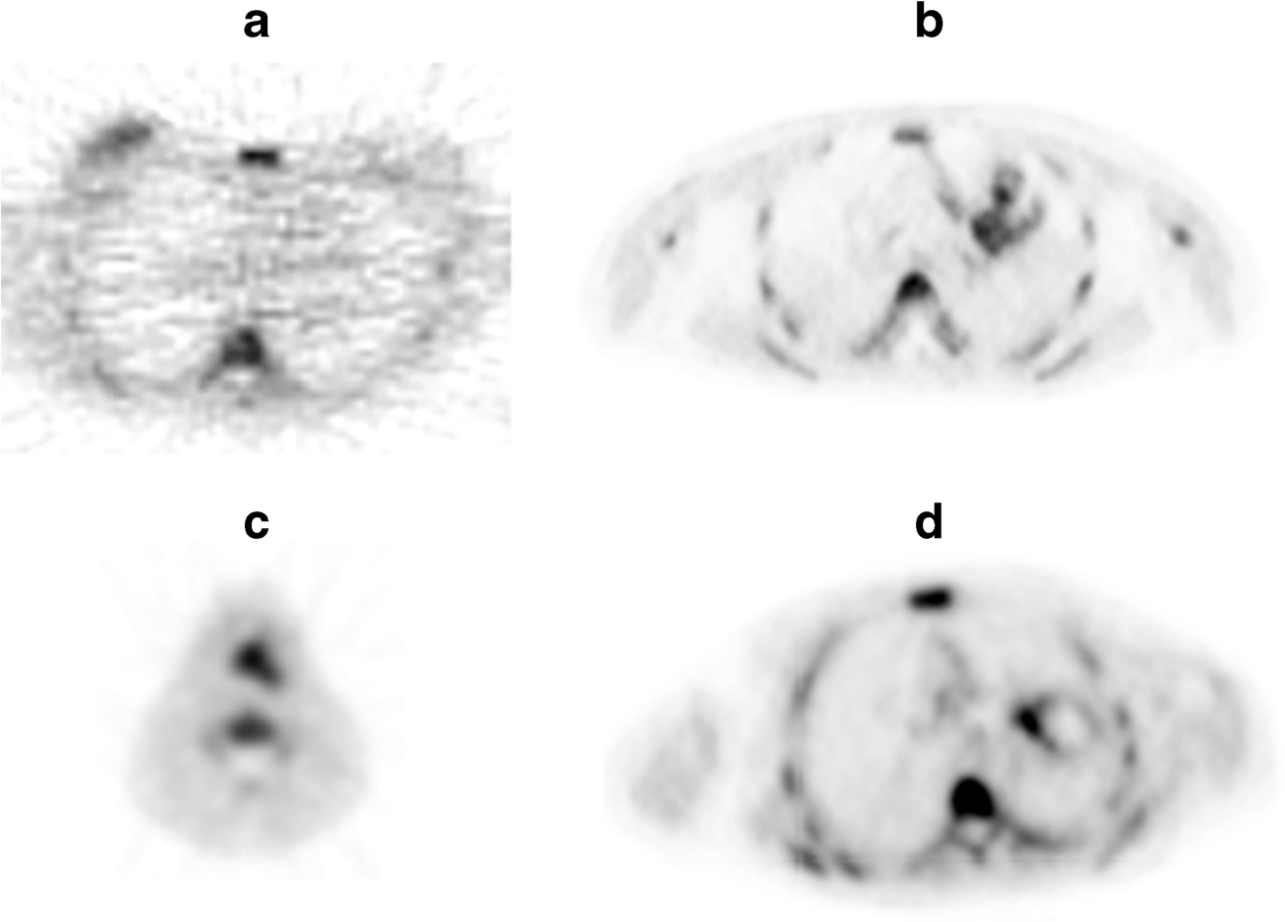
^18^F–FLT PET scan of all four cohorts. **a** Kenny et al. (breast); **b** Trigonis et al. (NSCLC); **c**, **d** de Langen et al., HNC and NSCLC, respectively

The literature search generated 1728 results: 630 in PubMed, 1076 in EMBASE.com and 22 in the Cochrane Library. No ongoing or unpublished trials were identified. After removing duplicates, 1172 references remained (Fig. 1). Out of 1172, four articles (five patient cohorts) were considered eligible [14–17]. We obtained permission to re-analyze the original ^18^F–FLT repeatability data from four of these cohorts, comprising data of 30 patients and 52 individual lesions (ten in breast cancer [14], nine in head and neck squamous cell carcinoma [15], and 33 in non-small cell lung cancer patients from two cohorts [15, 16]; Fig. 2). All patients were included in this individual patient data meta-analysis and no scans had to be excluded. An overview of the cohorts can be found in Table 1.

**Table 1:** Cohort and patient characteristics; median (range)

Characteristic	Kenny	de Langen		Trigonis
Cancer type	BC	NSCLC	HNC	NSCLC
Patients	8	9	6	7
Tumors	10	15	9	18
Scanner - Manufacturer - Model	Siemens ECAT/962 HR+	Siemens ECAT EXACT HR+	Siemens ECAT EXACT HR+	Siemens Biograph 6 Truepoint TrueV
Reconstruction	FBP	OSEM	OSEM	OSEM
Iteration	–	2	2	4
Subsets	–	16	16	21
Voxel size (mm^3^)	2.62 × 2.62 × 2.42	2.57 × 2.57 × 2.43	3.43 × 3.34 × 2.43	2.67 × 2.67 × 2.00
Static reconstruction - Scan interval (min) - Frames:	45–65 Averaged	45–60 Averaged	45–60 Averaged	45–60 Summed
Time between scans (days)	4.5 (2–9)	2 (1–6)	1 (1)	4 (2–6)
Weight (kg) - Test - Retest	61.6 (53–106) 61.9 (51.3–107)	71 (61–83) 71 (61–86.5)	77 (65–85) 77 (65–85)	81.5 (66–96.8) 81.4 (65.6–97)
Injected dose (MBq) - Test - Retest	369 (246–380) 312 (153–379)	385 (253–389) 365 (341–397)	375 (334–390) 376 (354–405)	289 (254–332) 328 (283–361)

*No significant differences were present between the test-and-retest scans for any of the studies (Wilcoxon signed-rank test

*BC* breast cancer, *NSCLC* non-small cell lung cancer, *HNC* head and neck cancer

### Repeatability

SUV metrics were lower in the lung cancer dataset from Trigonis et al. [16] compared to the other three datasets (average SUV_mean_: 2.4 vs. 3.5, respectively; *p* < 0.05). In addition, the SUV_max_ and SUV_peak_ values in the breast cancer dataset from Kenny et al. [14] were higher compared to those from de Langen et al. [15]. Proliferative volumes and TLU were significantly smaller in the HNC group and the NSCLC lesions in the dataset from Trigonis et al. [16] were also significantly smaller than in the de Langen et al. dataset [15]. Despite overall proliferative volumes of the retest scan being significantly larger than the test scans (MATV: 14.5 vs. 15.6 ml, *p* = 0.02), no differences were found between the SUV metrics from test-and-retest scans (Table 2). When assessed per site, a small but significant difference in proliferative volume and TLU was only found in the dataset from Trigonis et al. (mean difference −2.3 ml and −4.2 ml respectively, *p* < 0.01) [16].

**Table 2:** Mean ^18^F–FLT uptake values of different uptake metrics overall and per cohort

Quantitative tracer uptake measures	Overall		Kenny		de Langen						Trigonis	
			BC		Overall		NSCLC		HNC		NSCLC	
	Test (mean ± SD)	Retest (mean ± SD)	Test (mean ± SD)	Retest (mean ± SD)	Test (mean ± SD)	Retest (mean ± SD)	Test (mean ± SD)	Retest (mean ± SD)	Test (mean ± SD)	Retest (mean ± SD)	Test (mean ± SD)	Retest (mean ± SD)
SUV_max_	5.0 ± 2.4	4.9 ± 2.4	7.2 ± 3.4	6.7 ± 3.5	4.9 ± 1.7	4.8 ± 1.7	5.2 ± 1.5	4.9 ± 1.6	4.5 ± 2.0	4.5 ± 2.0	3.8 ± 1.5	4.0 ± 1.8
SUV_peak_	4.0 ± 2.1	3.9 ± 2.0	5.7 ± 3.0	5.3 ± 3.1	4.0 ± 1.6	4.0 ± 1.6	4.3 ± 1.5	4.1 ± 1.5	3.7 ± 1.8	3.7 ± 1.8	2.9 ± 1.2	2.9 ± 1.3
SUV_mean_	3.1 ± 1.5	3.0 ± 1.5	4.1 ± 2.3	3.8 ± 2.4	3.3 ± 1.2	3.2 ± 1.1	3.4 ± 1.0	3.3 ± 1.0	3.1 ± 1.4	3.1 ± 1.4	2.3 ± 0.9	2.4 ± 1.0
TLU	49 ± 66	51 ± 66	86 ± 40	88 ± 55	56 ± 83	56 ± 81	77 ± 100	76 ± 97	23 ± 18	23 ± 19	25 ± 32	30 ± 35
Volume	14 ± 16	16 ± 17	25 ± 25	26 ± 27	15 ± 17	15 ± 16	19 ± 20	20 ± 19	7.0 ± 4.7	6.8 ± 4.4	10 ± 8.9	12 ± 12

*BC* breast cancer, *NSCLC* non-small cell lung cancer, *HNC* head and neck cancer, *SUV* standardized uptake value, *TLU* total lesion uptake

**Fig. 3:**
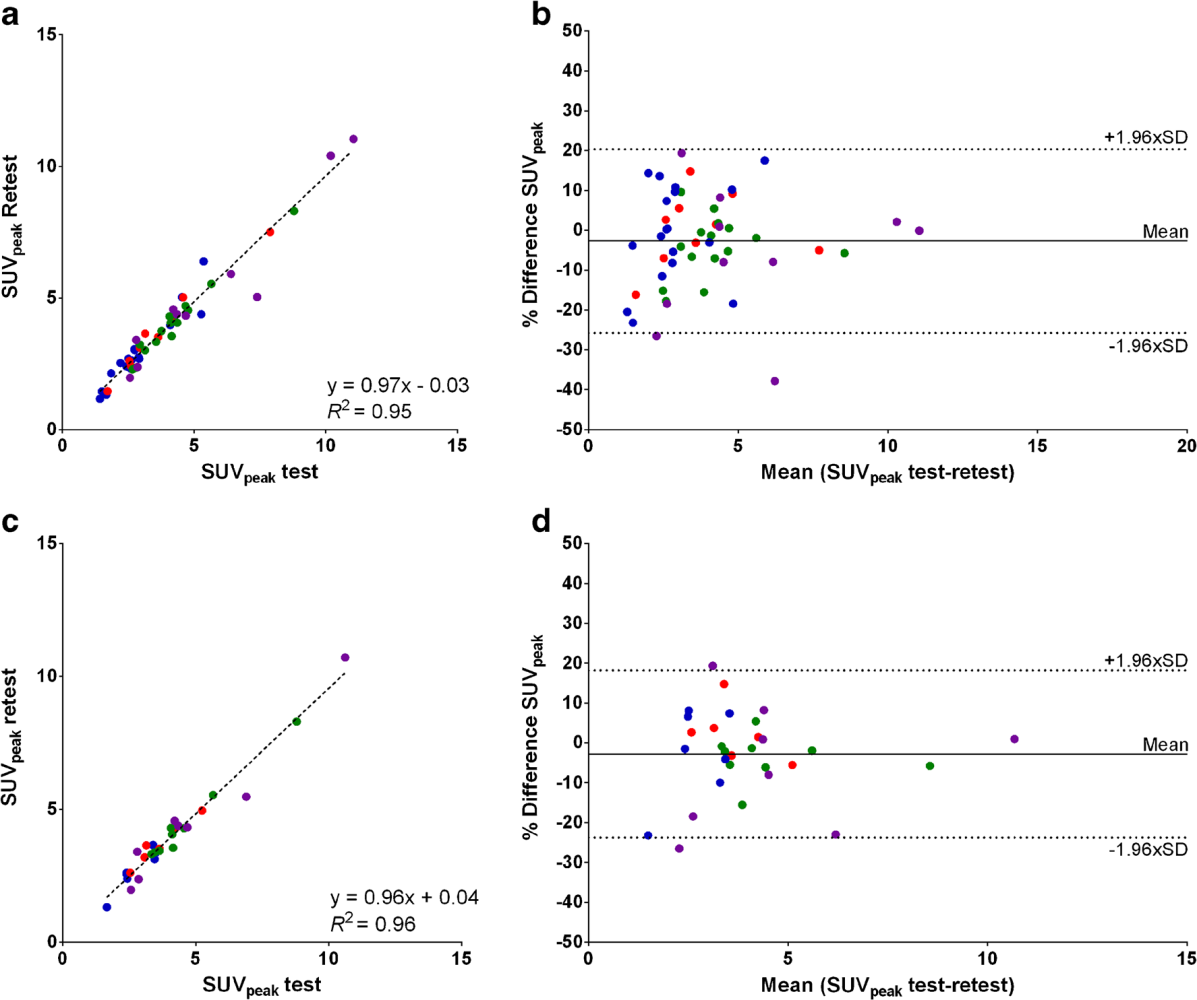
Test-and-retest SUV_peak_ plotted reciprocally per lesion (**a**) and per patient (**c**) with corresponding Bland–Altman plots (**b** and **d**, respectively). Similar patterns were seen for other SUV metrics. ( Trigonis;  de Langen [HNC];  de Langen [NSCLC];  Kenny)

Correlations between test-and-retest scans were strong for all uptake metrics per lesion as well as averaged per patient (*R*^2^ ≥ 0.93 and ICC ≥ 0.96, Fig. 3). Moreover, no systematic bias was present between both scans as revealed by the correlation plots (slope, 0.98–1.04, Fig. 3) and the Bland–Altman plots (Fig. 3). Overall, the best repeatability of quantitative ^18^F–FLT PET/CT was obtained using SUV_peak_ (RCs 23.1%, Table 3). No differences in RCs were found between the individual SUV metrics.

**Table 3:** Mean relative differences and RCs on lesion level for several uptake metrics

Quantitative tracer uptake measures	Overall		Kenny		de Langen						Trigonis	
			BC		Overall		NSCLC		HNC		NSCLC	
	Mean difference (%)	RC (%)	Mean difference (%)	RC (%)	Mean difference (%)	RC (%)	Mean difference (%)	RC (%)	Mean difference (%)	RC (%)	Mean difference (%)	RC (%)
SUV_max_	−3.14	25.54	−9.05	25.86	−3.38	19.26	−5.60	19.80	0.32	16.91	0.47	31.13
SUV_peak_	−2.72	23.06	−6.83	33.22	−2.56	16.42	−4.24	14.96	0.24	18.16	−0.65	24.29
SUV_mean_	−3.32	25.16	−12.62	41.89	−1.40	14.42	−2.80	13.01	0.93	16.24	−0.72	21.12
TLU	3.70	36.38	−5.43	37.03	0.06	27.30	1.69	29.75	−2.65	23.32	12.09	41.88
Volume	5.43	35.95	−0.01	40.64	1.45	24.35	4.47	27.46	−3.59	14.47	12.84	43.68

*BC* breast cancer, *NSCLC* non-small cell lung cancer, *HNC* head and neck cancer, *SUV* standardized uptake value, *TLU* total lesion uptake

**Fig. 4:**
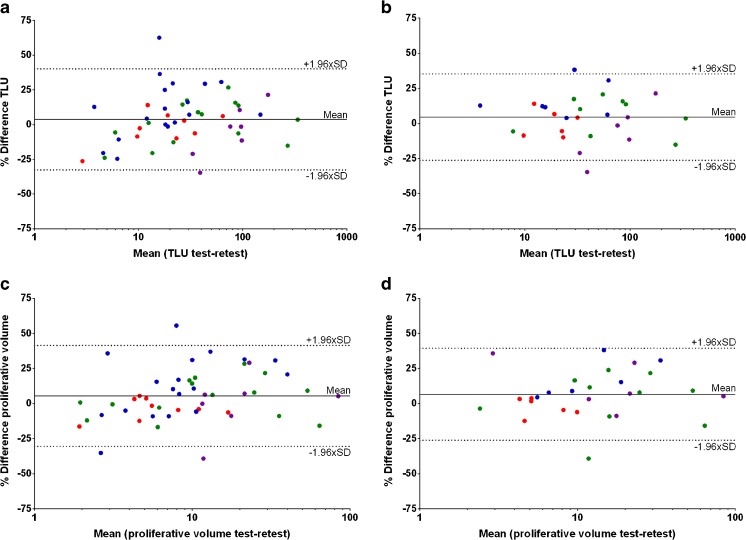
Bland–Altman plots of total lesion uptake (TLU) and proliferative volume on lesion (**a** and **c**, respectively) and patient level (**b** and **d**, respectively). ( Trigonis;  de Langen [HNC];  de Langen [NSCLC];  Kenny)

Variability of proliferative volume and TLU (RCs 36.0 and 36.4%, respectively) were significantly worse than for SUV metrics, with an average increase in RC of 9.6 ± 6.6% (*p* ≤ 0.02)(Fig. 4). When the datasets were evaluated individually, variability of SUV_peak_ and SUV_mean_ within the de Langen et al. [15] cohorts was significantly smaller compared to those of the breast cancer dataset, the only one reconstructed with FBP (*p* < 0.02) [14]. In general, the largest variability was seen in the latter dataset. When comparing only the OSEM reconstructed datasets, RCs for SUV_max_, SUV_peak_, and SUV_mean_ changed to 25, 20, and 17% respectively, but RCs of proliferative volumes and TLU remained close to 35%. An overview of the absolute repeatability coefficients for each quantitative uptake metric can be found in supplemental Tables 4 and 5.

Assessment of repeatability on a patient level improved repeatability in general (Table 4). Improvement of repeatability weighted for lesions number was < 2% compared to unweighted averaging per patient. For the SUV metrics, a decrease in RC was largest in the de Langen dataset [15]. Only SUV_mean_ showed a slight increase in variability, which was caused by one lesion with a 53% difference (4 SDs) between both scans from the breast cancer dataset. If excluded, repeatability of SUV_mean_ improved to 19%, while other SUV metrics remained unaffected. RCs of proliferative volume and TLU also decreased to < 30%, with the exception of the breast dataset [14].

**Table 4:** Mean relative differences and RCs on patient level for several uptake metrics

Quantitative tracer uptake measures	Overall		Kenny		de Langen						Trigonis	
			BC		Overall		NSCLC		HNC		NSCLC	
	Mean difference (%)	RC (%)	Mean difference (%)	RC (%)	Mean difference (%)	RC (%)	Mean difference (%)	RC (%)	Mean difference (%)	RC (%)	Mean difference (%)	RC (%)
SUV_max_	−3.54	20.63	−8.61	21.82	−1.82	14.92	−4.27	14.47	1.85	13.59	−1.43	28.31
SUV_peak_	−2.76	21.00	−5.81	31.50	−1.32	13.21	−3.74	11.07	2.31	13.82	−2.37	22.34
SUV_mean_	−3.99	26.44	−12.14	43.26	−0.18	10.93	−1.72	9.06	2.14	12.72	−2.83	20.79
TLU	4.51	30.85	−7.10	38.73	3.52	22.83	5.75	25.26	0.18	18.79	16.58	25.22
Volume	6.67	32.81	−0.52	44.24	3.23	23.60	6.96	26.95	−2.36	12.53	20.19	27.98

*BC* breast cancer, *NSCLC* non-small cell lung cancer, *HNC* head and neck cancer, *SUV* standardized uptake value, *TLU* total lesion uptake

### Lesion selection

**Fig. 5:**
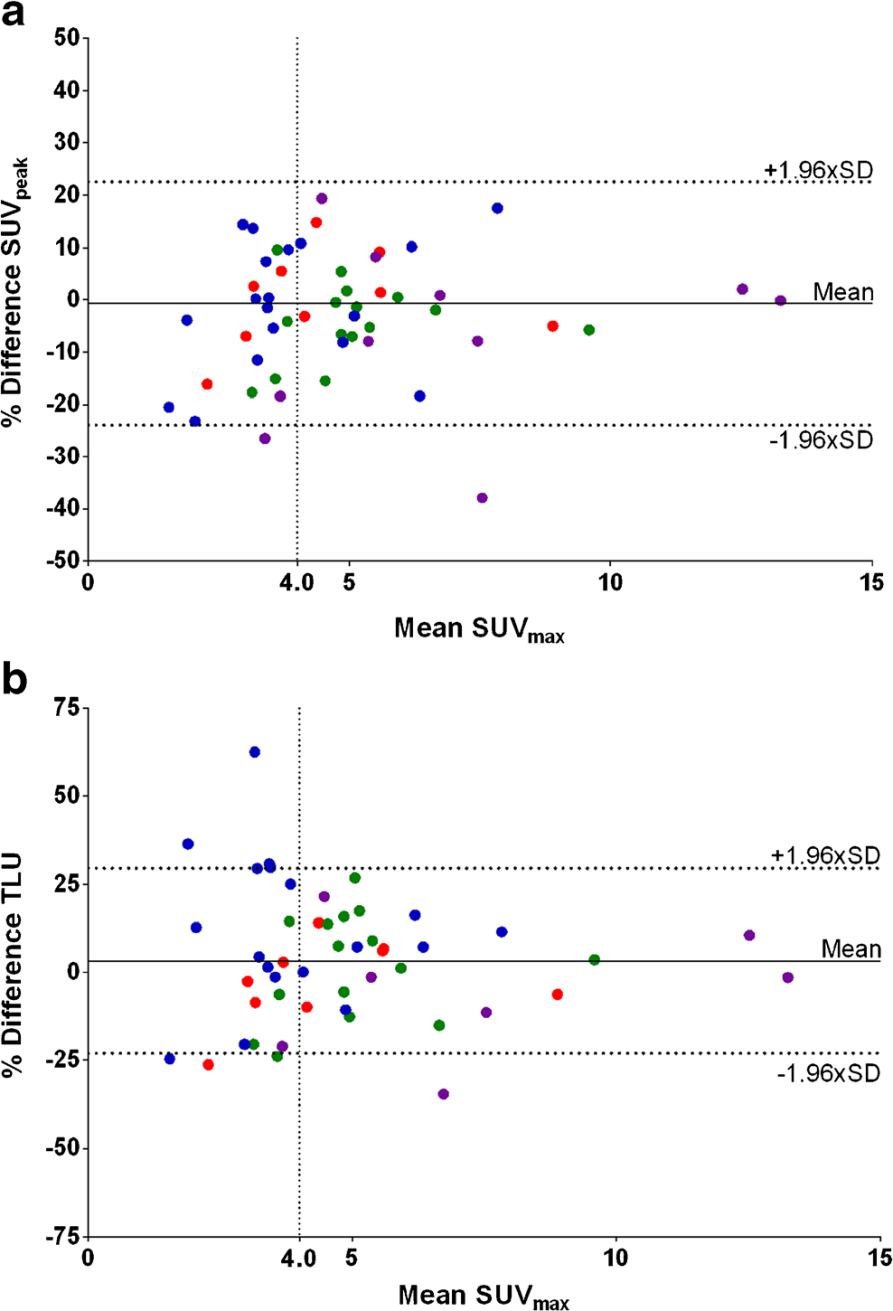
Variability of SUV_peak_ (**a**) and proliferative volume (**b**) plotted against SUV_max_. The *dashed horizontal lines* indicate the cut-off values used for the lesions selection strategies. ( Trigonis;  de Langen [HNC];  de Langen [NSCLC];  Kenny)

Assessing repeatability including only lesions with SUV_max_ ≥ 4.0 decreased variability of volumetric metrics but did not influence RCs of SUV metrics (RCs 26–28%, Fig. 5). The former is mainly caused by a large decrease of RCs in the Trigonis dataset (−20%). If only lesions larger than 4.2 ml were included in the analysis, no significant change in variability of SUV, proliferative volume or TLU was seen (RCs 22–25% and 34–36%, respectively). Similar results were observed when only the hottest or primary lesions were assessed. Combining the two selection criteria SUV_max_ ≥ 4.0 and lesions ≥ 4.2 ml did not further improve results. No significant change in repeatability of SUV metrics was seen when analyzing cohorts individually. In addition, applying lesions selection criteria to the per-patient analysis did not decrease variability of SUV and volumetric ^18^F–FLT uptake measures.

### Normalization to blood uptake

Overall, repeatability deteriorated significantly when TBR was used (RCs +49–52%; *p* < 0.02). The effect on the HNC dataset using the carotid artery was not different compared to the lung cancer datasets using the larger ascending aorta. In particular, repeatability of the breast dataset worsened by calculating the TBR, showing an increase of > 50% for all metrics. This is likely explained by the variability of the bloodpool SUV being significantly larger in the FBP reconstructed dataset compared to the OSEM reconstructed datasets (SD: 34 vs. 13%). When this cohort was excluded, RCs of TBR metrics were no longer significantly different from the SUV metrics.

## Discussion

This individual patient data meta-analysis combined available data from four different ^18^F–FLT PET test–retest cohorts acquired in three different cancer types at three different centers. Of the quantitative ^18^F–FLT uptake measures commonly used in oncological setting, SUV metrics showed better repeatability overall than the volumetric metrics. Unfortunately, we did not obtain permission from one study to re-analyze their data [17]. However, individual SUV_max_, SUV_peak_, and SUV_mean_ values were reported in this article. If these numbers are included in the analysis, RCs of the SUV metrics improve by approximately 2%, yet do not influence the results significantly.

If we compare our results to those published in the original reports, similar variability was found for SUV_max_ [15, 16]. Repeatability of SUV_mean_ improved when threshold based segmentation was applied for the Trigonis et al. [16] cohort (RC: 29.8 vs. 21.1). In contrast, variability of SUV_mean_ increased in the FBP dataset compared to manual delineation (RC: 20.6 vs. 41.9) [14]. This is also seen when other segmentation algorithms are used for lesion delineation in this FBP reconstructed dataset and raises the issue of appropriateness of semi-automatic segmentation in FBP reconstructed images [21]. Unfortunately, the raw data of this dataset were not available, so no reconstruction using OSEM could be performed.

The repeatability of ^18^F–FLT SUV metrics from this study is better than the 30% threshold suggested by PET response criteria in solid tumors (PERCIST) for ^18^F–FDG PET. The repeatability is similar to that found in a recent prospective multi-center study (*n* = 10 patients, one lesion per patient; five institutions) on ^18^F–FLT in gliomas (RCs 19–23%) [22]. In addition, our results are in line with multiple other single-center repeatability studies for several different tracers [12, 23, 24]. In general, multi-institutional studies yield higher variability (RCs 28–47%) [10, 11, 13]. The lower variability found in this study might be partly explained by the fact that data were acquired in strictly controlled single-center setting. Moreover, no differences in uptake time between the test and retest scans were present because static images were generated from dynamic scans. This removed the variability in uptake time on SUV that is typically encountered when acquiring static images. However, a previous study has shown that ^18^F–FLT tumor uptake reached equilibrium at 30 min post injection in NSCLC [19].

Several other studies also found poorer repeatability of volumetric metrics compared to SUV metrics (RCs > 30%) [12, 18]. In our study, VOIs were defined using semi-automatic segmentation to minimize user dependency. In two out of three original reports, manual delineation was used, potentially contributing to the observed differences [14, 16]. It was expected that repeatability of volumetric metrics would be slightly worse in the FBP dataset due to higher noise levels and streak artifacts. In contrast to our expectation, PET/CT data showed a higher variability of proliferative volume and TLU compared to PET only data. Moreover, variability of proliferative volume was larger in our study compared to the original report for the PET/CT data (RCs 43.7 vs. 30.6%) [16]. This discrepancy was mainly caused by low ^18^F–FLT uptake of lesions in the PET/CT dataset, resulting in low tumor-to-background ratios. As semi-automatic segmentation methods require adequate contrast between tumor and background radioactivity, accurate VOI definition can be compromised. This is supported by the fact that results significantly improve when including only lesions with SUV_max_ > 4.0.

Two studies validating simplified quantitative metrics of ^18^F–FLT uptake in NSCLC showed a stronger correlation of TBR with the uptake constant K_i_ (estimated from kinetic analysis) compared to SUV [19, 25]. In our study, we found that normalizing SUV to blood pool radioactivity concentrations significantly increases variability for ^18^F–FLT images reconstructed with FBP. Moreover, TBR has been shown to be highly time dependent for ^18^F–FLT, limiting its use in response assessment, especially in busy clinical settings [19, 26].

It is suggested that assessment of response per patient rather than per lesion may improve correlation with patient outcome [27]. Similar to other studies, assessing repeatability per patient improved RCs by reducing the non-systematic differences between the test-and-retest scans. To our knowledge, only one study has been performed comparing response assessment per patient and per lesion [28]. Here, no significant differences in performance of the two methods were found. Yet, in this ^18^F–FDG study, the same threshold of 30% to differentiate between stable disease and progressive disease or partial response was used for both methods [28]. We therefore propose that future response assessment studies with ^18^F–FLT PET/CT should also assess the response per patient, while taking the per-patient variability into account.

In the current study, we have used symmetric limits to assess repeatability of quantitative ^18^F–FLT uptake metrics. Symmetrical RCs are commonly used in PET repeatability literature, however recent papers have discussed their applicability in daily clinical practice [10, 29]. In test–retest studies, often no golden standard is available and therefore relative differences are calculated using the average of the two measurements. This differs from response assessment in clinical setting where change is determined relative to a single baseline value and therefore asymmetrical RCs are suggested to be more suitable. If we calculate asymmetric RCs at lesion level, the overall upper (URC) and lower limits (LRC) of the RCs are: SUV_max_ (URC: 29.4%; LRC: -22.7%); SUV_mean_ (URC: 29.0%; LRC: -22.5%); SUV_peak_ (URC: 26.0%; LRC -20.6%); TLU (URC: 44.6%; LRC -30.9%); and volume (URC: 43.7%; LRC: -30.4%). These results show a slight shift in RCs of SUV metrics compared to the symmetric limits, however remain within 30%. On a patient level, asymmetrical RCs improved RCs of SUV: SUV_max_ (URC: 21.1%; LRC: -18.3%); SUV_mean_ (URC: 15.3%; LRC: -23.3%); SUV_peak_ (URC: 16.8%; LRC -18.8%); TLU (URC: 34.1%; LRC -27.9%); and volume (URC: 36.3%; LRC: -28.7%).

The use of different PET scanners and the heterogeneity in reconstruction methods between cohorts could have contributed to the variability in the uptake and volumetric metrics. However, despite these limitations, repeatability of ^18^F–FLT was better compared to several other standardized multi-center studies that prospectively evaluated repeatability of ^18^F–FDG. In contrast to other meta-analyses, we increased robustness by re-analyzing all scans and thus minimizing variability due to data analysis and allowing direct comparison of quantitative uptake metrics. To date, this individual patient data meta-analysis provides the largest test–retest ^18^F–FLT PET cohort. These results should ideally be confirmed in a large prospective multi-center PET/CT study.

## Conclusions

In this multi-center, individual patient data meta-analysis, we found that repeatability of ^18^F–FLT tumor uptake is comparable to that of ^18^F–FDG PET/CT. In multi-center studies, a 25% and 20% difference in individual ^18^F–FLT SUV metrics likely represents a true change in tumor uptake at lesion and patient level, respectively. In case of volumetric measurements, higher thresholds are needed compared to SUV metrics, especially for lesions with SUV_max_ < 4.0 at baseline.

## Electronic supplementary material

ESM 1 (DOCX 28 kb)
