# Supplementary material for: Repeatability of quantitative 18F-FLT uptake measurements in solid tumors: an individual patient data multi-center meta-analysis
Source: Eur J Nucl Med Mol Imaging. 2018 Jan 23;45(6):951–61. doi: 10.1007/s00259-017-3923-x (PMC5915500; doi:10.1007/s00259-017-3923-x)
Supplement: Supplementary file 1 — (DOCX 28 kb) [file 259_2017_3923_MOESM1_ESM.docx]

**Supplemental data:**

Supplemental table 1. Search strategy in Pubmed October 20^th^ , 2016 (read from bottom-up).

| **Search #3 NOT (animals[mh] NOT humans[mh])** | **630** |
| --- | --- |
| **Search #1** AND **#2** | **699** |
| Search **neoplasms[mesh]** OR **cancer[sb]** OR **oncolog*[tiab]** OR **cancer*[tiab]** OR **neoplas*[tiab]** OR **tumour*[tiab]** OR **tumor[tiab]** OR **tumors[tiab]** OR **tumori*[tiab]** OR **carcinom*[tiab]** OR **melanom*[tiab]** OR **lymphom*[tiab]** OR **leukemi*[tiab]** OR **malignan*[tiab]** OR **metasta*[tiab]** OR **carcinogen*[tiab]** OR **oncogen*[tiab]** OR **anticarcinogen*[tiab]** OR **sarcoma*[tiab]** OR **precancerous[tiab]** OR **paraneoplastic[tiab]** OR **neuroma*[tiab]** OR **blastoma*[tiab]** OR **meningioma*[tiab]** OR **lymphangioma*[tiab]** OR **lymphangiomyoma*[tiab]** OR **lymphangiosarcoma*[tiab]** OR **"hodgkin disease"[tiab]** OR **plasmacytoma*[tiab]** OR **carcinosarcoma*[tiab]** OR **hepatoblastoma*[tiab]** OR **mesenchymoma*[tiab]** OR **chordoma*[tiab]** OR **germinoma*[tiab]** OR **gonadoblastoma*[tiab]** OR **mesonephroma*[tiab]** OR **teratoma*[tiab]** OR **teratocarcinoma*[tiab]** OR **nsclc[tiab]** | **5481558** |
| Search **("alovudine"[Supplementary Concept]** OR **alovudine[tiab]** OR **FDDT[tiab]** OR **18F-FLT[tiab]** OR **(18)FLT cpd[tiab]** OR **(18F)FLT[tiab]** OR **fluorothymidin*[tiab]** OR **FDDT[tiab]** OR **deoxythymidin*[tiab]** OR **dideoxythymidin*[tiab]** OR **fluordeoxythymidin*[tiab]** OR **FLT[tiab]** OR **FLTs[tiab]) AND ("Positron-Emission Tomography"[Mesh]** OR **pet[tiab]** OR **pet/*[tiab]** OR **petscan*[tiab]** OR **(emission[tiab]** AND **(tomograph [tiab]** OR **tomographs [tiab]** OR **tomographic*[tiab]** OR **tomography[tiab]** OR **tomographies[tiab]** OR **scan[tiab])))** | **739** |

Supplemental table 2. Search strategy in Embase October 20^th^ , 2016 (read from bottom-up).

| No. | Query | Results |
| --- | --- | --- |
| #4 | **#3** AND [humans]/lim | **1076** |
| #3 | **#1** AND **#2** | **1347** |
| #2 | **'neoplasm'**/exp OR **oncolog***:ti,ab OR **cancer***:ti,ab OR **neoplas***:ti,ab OR **tumour***:ti,ab OR **tumor**:ti,ab OR **tumors**:ti,ab OR **tumori***:ti,ab OR **carcinom***:ti,ab OR **melanom***:ti,ab OR **lymphom***:ti,ab OR **leukemi***:ti,ab OR **malignan***:ti,ab OR **metasta***:ti,ab OR **carcinogen***:ti,ab OR **oncogen***:ti,ab OR **anticarcinogen***:ti,ab OR **sarcoma***:ti,ab OR **precancerous**:ti,ab OR **paraneoplastic**:ti,ab OR **neuroma***:ti,ab OR **blastoma***:ti,ab OR **meningioma***:ti,ab OR **lymphangioma***:ti,ab OR **lymphangiomyoma***:ti,ab OR **lymphangiosarcoma***:ti,ab OR **'hodgkin disease'**:ti,ab OR **plasmacytoma***:ti,ab OR **carcinosarcoma***:ti,ab OR **hepatoblastoma***:ti,ab OR **mesenchymoma***:ti,ab OR **chordoma***:ti,ab OR **germinoma***:ti,ab OR **gonadoblastoma***:ti,ab OR **mesonephroma***:ti,ab OR **teratoma***:ti,ab OR **teratocarcinoma***:ti,ab OR **nsclc**:ti,ab | **4717398** |
| #1 | **'3`fluorothymidine'**/exp OR **alovudine**:ti,ab OR **'18f flt'**:ti,ab OR **'flt'**:ti,ab OR **fluorothymidin***:ti,ab OR **fddt**:ti,ab OR **deoxythymidin***:ti,ab OR **dideoxythymidin***:ti,ab OR **fluordeoxythymidin***:ti,ab OR **flt**:ti,ab OR **flts**:ti,ab AND (**'positron emission tomography'**/exp OR **pet**:ti,ab OR **petscan***:ti,ab OR (**emission**:ti,ab AND (**tomograph**:ti,ab OR **tomographs**:ti,ab OR **tomographic***:ti,ab OR **tomography**:ti,ab OR **tomographies**:ti,ab OR **scan**:ti,ab))) | **1467** |

Supplemental table 3. Search strategy in Cochrane October 20^th^ , 2016 (read from bottom-up).

| No. | Query | Results |
| --- | --- | --- |
| #3 | **#1** AND **#2** | **23** |
| #2 | **oncolog* or cancer* or neoplas* or tumour* or tumor or tumors or tumori* or carcinom* or melanom* or lymphom* or leukemi* or malignan* or metasta* or carcinogen* or oncogen* or anticarcinogen* or sarcoma* or precancerous or paraneoplastic or neuroma* or blastoma* or meningioma* or lymphangioma* or lymphangiomyoma* or lymphangiosarcoma* or "hodgkin disease" or plasmacytoma* or carcinosarcoma* or hepatoblastoma* or mesenchymoma* or chordoma* or germinoma* or gonadoblastoma* or mesonephroma* or teratoma* or teratocarcinoma* or nsclc:ti,ab,kw (Word variations have been searched)** | **127840** |
| #1 | **(alovudine or FDDT or "18F-FLT" or "FLT" or fluorothymidin* or FDDT or deoxythymidin* or dideoxythymidin* or fluordeoxythymidin* or FLTs) and ("Positron-Emission Tomography" or pet or petscan* or (emission and (tomograph or tomographs or tomographic* or tomography or tomographies or scan))):ti,ab,kw (Word variations have been searched)** | **23** |

Supplemental table 4. Mean Absolute Differences and RCs on lesion level for Several Uptake Metrics

| *Quantitative tracer uptake measures* | *Overall* | | *Kenny* | | *de Langen* | | | | | | *Trigonis* | |
| --- | --- | --- | --- | --- | --- | --- | --- | --- | --- | --- | --- | --- |
|  |  |  | *BC* | | *Overall* | | *NSCLC* | | *HNC* | | *NSCLC* | |
|  | *Mean difference (%)* | *RC (%)* | *Mean difference (%)* | *RC (%)* | *Mean difference (%)* | *RC (%)* | *Mean difference (%)* | *RC (%)* | *Mean difference (%)* | *RC (%)* | *Mean difference (%)* | *RC (%)* |
| SUV_max_ | -0.11 | 1.26 | -0.52 | 1.67 | -0.13 | 0.78 | -0.24 | 0.79 | 0.04 | 0.66 | 0.14 | 1.37 |
| SUV_peak_ | -0.09 | 0.93 | -0.31 | 1.61 | -0.09 | 0.56 | -0.16 | 0.51 | 0.03 | 0.59 | 0.03 | 0.80 |
| SUV_mean_ | -0.08 | 0.74 | -0.37 | 1.27 | -0.03 | 0.43 | -0.08 | 0.38 | 0.04 | 0.50 | 0.02 | 0.55 |
| TLU | 2.24 | 20.31 | 1.88 | 34.32 | 0.86 | 21.04 | 1.29 | 26.77 | 0.16 | 3.91 | 4.23 | 11.14 |
| Volume | 1.14 | 6.57 | 1.04 | 7.32 | 0.34 | 6.15 | 0.69 | 7.77 | -0.25 | 0.83 | 2.25 | 6.58 |
| *BC=breast cancer; NSCLC=non-small cell lung cancer; HNC=head and neck cancer; SUV=standardized uptake value; TLU=total lesion uptake* | | | | | | | | | | | | |

Supplemental table 5. Mean Absolute Differences and RCs on patient level for Several Uptake Metrics

| *Quantitative tracer uptake measures* | *Overall* | | *Kenny* | | *de Langen* | | | | | | *Trigonis* | |
| --- | --- | --- | --- | --- | --- | --- | --- | --- | --- | --- | --- | --- |
|  |  |  | *BC* | | *Overall* | | *NSCLC* | | *HNC* | | *NSCLC* | |
|  | *Mean diffference* | *RC* | *Mean diffference* | *RC* | *Mean diffference* | *RC* | *Mean diffference* | *RC* | *Mean diffference* | *RC* | *Mean diffference* | *RC* |
| SUV_max_ | -0.17 | 0.89 | -0.44 | 1.13 | -0.11 | 0.69 | -0.22 | 0.68 | 0.06 | 0.61 | 0.01 | 0.84 |
| SUV_peak_ | -0.11 | 0.77 | -0.22 | 1.25 | -0.08 | 0.53 | -0.18 | 0.49 | 0.06 | 0.51 | -0.03 | 0.49 |
| SUV_mean_ | -0.10 | 0.66 | -0.31 | 1.08 | -0.02 | 0.38 | -0.07 | 0.35 | 0.05 | 0.40 | -0.04 | 0.37 |
| TLU | 2.19 | 25.33 | 1.49 | 36.87 | 0.86 | 25.30 | 1.43 | 33.31 | 0.00 | 3.25 | 5.63 | 13.74 |
| Volume | 1.27 | 7.41 | 1.15 | 7.97 | 0.48 | 7.19 | 0.92 | 9.38 | -0.19 | 0.72 | 3.07 | 7.28 |
| *BC=breast cancer; NSCLC=non-small cell lung cancer; HNC=head and neck cancer; SUV=standardized uptake value; TLU=total lesion uptake* | | | | | | | | | | | | |
